# Deep Learning Model for Automated Segmentation of Orbital Structures in MRI Images

**DOI:** 10.1007/s00062-025-01535-2

**Published:** 2025-06-26

**Authors:** Esmira Bakhshaliyeva, Lara Noelle Reiner, Moudather Chelbi, Jawed Nawabi, Anna Tietze, Michael Scheel, Mike Wattjes, Andrea Dell’Orco, Aymen Meddeb

**Affiliations:** 1 Department of Radiology, Charité Universitätsmedizin Berlin, Berlin, Germany; 2 Department of Neuroradiology, Charité—Universitätsmedizin Berlin/Berlin Institute of Health, Charitéplatz 1, 10117 Berlin, Germany; 3 Department of Neuroradiology, Hopital Maison Blanche, CHU Reims, Champagne-Ardenne, France; 4 BIH Biomedical Innovation Academy, Berlin Institute of Health at Charité—Universitätsmedizin Berlin, Berlin, Germany

**Keywords:** Segmentation, Deep learning, MRI, Ophthalmology, NnU-Net

## Abstract

**Background:**

Magnetic resonance imaging (MRI) is a crucial tool for visualizing orbital structures and detecting eye pathologies. However, manual segmentation of orbital anatomy is challenging due to the complexity and variability of the structures. Recent advancements in deep learning (DL), particularly convolutional neural networks (CNNs), offer promising solutions for automated segmentation in medical imaging. This study aimed to train and evaluate a U-Net-based model for the automated segmentation of key orbital structures.

**Methods:**

This retrospective study included 117 patients with various orbital pathologies who underwent orbital MRI. Manual segmentation was performed on four anatomical structures: the ocular bulb, ocular tumors, retinal detachment, and the optic nerve. Following the UNet autoconfiguration by nnUNet, we conducted a five-fold cross-validation and evaluated the model’s performances using Dice Similarity Coefficient (DSC) and Relative Absolute Volume Difference (RAVD) as metrics.

**Results:**

nnU-Net achieved high segmentation performance for the ocular bulb (mean DSC: 0.931) and the optic nerve (mean DSC: 0.820). Segmentation of ocular tumors (mean DSC: 0.788) and retinal detachment (mean DSC: 0.550) showed greater variability, with performance declining in more challenging cases. Despite these challenges, the model achieved high detection rates, with ROC AUCs of 0.90 for ocular tumors and 0.78 for retinal detachment.

**Conclusions:**

This study demonstrates nnU-Net’s capability for accurate segmentation of orbital structures, particularly the ocular bulb and optic nerve. However, challenges remain in the segmentation of tumors and retinal detachment due to variability and artifacts. Future improvements in deep learning models and broader, more diverse datasets may enhance segmentation performance, ultimately aiding in the diagnosis and treatment of orbital pathologies.

## Introduction

Magnetic resonance imaging (MRI) is a valuable diagnostic imaging technique in the visualization and assessment of orbital anatomy and pathology detection detection [1, 2]. The high soft tissue contrast and spatial resolution aids for an accurate diagnosis and treatment planning purposes of conditions in various disease entities including ocular tumors [3], optic nerve diseases [4], and retinal detachments [5]. The complexity of orbital anatomy in combination with the variable presentation of diseases, makes manual segmentation time-consuming, subject to inter-rater variability, and potentially inconsistent.

Accurate segmentation of ocular structures is important for the quantification of disease burden and for treatment planning. In particular, radiotherapy planning for ocular tumors requires precise delineation of tumor boundaries is critical for effective radiation targeting while minimizing damage to healthy tissues [6, 7]. Detection of inflammation in the optic nerve, such as in optic neuritis, relies on accurate segmentation to assess inflammation extent and guide treatment strategies [8]. Additionally, automated segmentation and detection of periorbital phlegmons and abscesses could significantly enhance surgical planning and antibiotic therapy optimization [9]. Thus, reliable segmentation algorithms are essential tools that enhance diagnostic accuracy and improve patient outcomes in these diverse clinical scenarios.

Advancements in deep learning (DL) have provided promising directions for automating the segmentation process, improving accuracy, and reducing analysis time. Among various DL approaches, convolutional neural networks (CNNs) have emerged as a powerful tool for medical image analysis [10–13]. The nnU-Net (no-new-UNET) framework [14], designed as a self-adapting approach to U‑Net-based architectures, represents an innovative step forward, offering an out-of-the-box solution for medical image segmentation challenges.

This study aims to evaluate the capability and effectiveness of the nnU-Net in segmenting simultaneously diverse orbital components, including ocular bulb, ocular tumors, retinal detachment and the optic nerve on MRI scans. Utilizing a patient cohort with various pathologies, we sought to train and validate an nnU-Net model capable of handling the complexity and diversity of orbital diseases.

## Materials and Methods

### Study Population and MRI Sequences

**Fig. 1:**
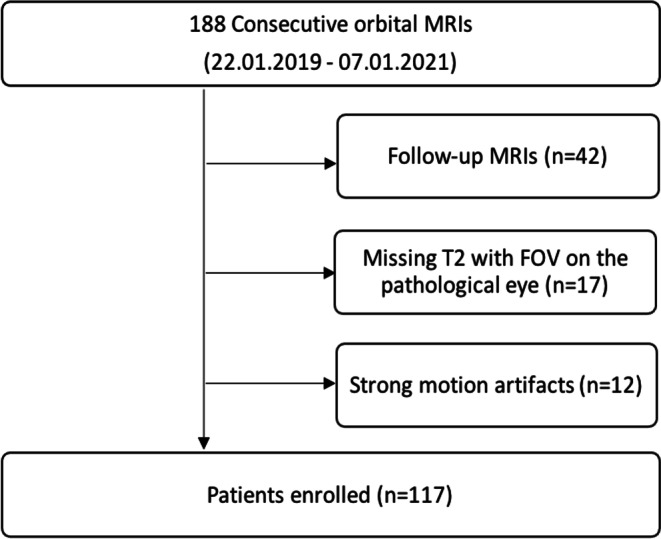
Patient Selection Process for Orbital MRI Segmentation Study

In this retrospective study, orbital MRI scans were collected from a tertiary hospital, with approval from the ethics committee (No. EA4/136/21). Due to the study’s retrospective design, the requirement for informed consent was waived. All patient data were anonymized and handled in compliance with the Declaration of Helsinki and applicable data protection regulations to ensure patient privacy. Orbital MRI scans were conducted between January 22, 2019, and January 7, 2021. During this period, 188 consecutive orbital MRI scans were reviewed. Scans that were follow-up examinations, lacked T2-weighted sequences for the affected eye, or contained significant artifacts were excluded from the analysis. The patients underwent an institution-specific orbital MRI protocol, which included mono-orbital transverse T1 and T2 turbo spin-echo (TSE) sequences, a diffusion-weighted imaging (DWI) sequence with an apparent diffusion coefficient (ADC) map, a coronal T2 TSE with fat suppression (FS), and a transverse T1 volumetric interpolated breath-hold examination (VIBE) with fat suppression following contrast media injection. The detailed selection process is outlined in Fig. 1.

### Manual Segmentation

Manual segmentation was performed using a mono-orbital 2D transverse turbo spin-echo T2-weighted sequence. The sequence parameters included an echo time (TE) of 69 ms and a repetition time (TR) of 3500 ms. The field of view (FOV) was 64 × 79 mm, and the matrix size was 156 × 192. The T2 sequences were manually segmented by a radiology resident with four years of experience and a board-certified radiologist with seven years of experience. This process utilized 3D-Slicer software version 5.2.1, which allows for the precise delineation of the orbit, optic nerve, and, where applicable, any tumors or retinal detachments. These segmentations served as the ground truth for the study. Each pixel in the resulting 2D binary masks was classified according to its anatomical or pathological category.

### Deep-learning Model

The nnU-Net framework (Version 2) was utilized for this study due to its “plug-and-play” functionality [14]. A five-fold cross-validation strategy was implemented, in which the subject list was randomly shuffled and divided into five datasets according to the nnU-Net directory structure.

The training employed the 3d_fullres UNet architecture including the fold‑1 option reserving 20% of the training data for validation and resulting in a final train:validation:test split ratio of 64:16:20. All models were trained for 1000 epochs. Following training and segmentation of the test datasets, metrics were calculated using custom Python scripts, incorporating libraries such as NumPy [15], NIBabel [16], and Pandas [17]. The Dice Similarity Coefficient (DSC) was calculated for each ground truth and prediction mask as outlined in Eq. 1, with volumetric measurements determined by voxel counting and converted multiplying for the voxel volume. A significant effect of fold on DSC was excluded through testing with ANOVA (formula DSC ~ fold number) all orbital structures.

### Statistical Analysis and Evaluation

This study employed two established metrics for evaluating segmentation quality: the Dice Similarity Coefficient (DSC) and the Relative Absolute Volume Difference (RAVD). The DSC measures the overlap between segmented volumes and the ground truth, calculated as follows:

$$DSC=\frac{2\times true\,\textit{positive}\,\textit{voxels}}{\begin{array}{c}2\times true\,\textit{positive}\,\textit{voxels}+\textit{false}\,\textit{positive}\,\textit{voxels}\\{}+\textit{false}\,\textit{negative}\,\textit{voxels}\end{array}}$$

A DSC value of 1 signifies perfect segmentation, while 0 indicates no overlap. Usually, a segmentation achieving a DSC higher than 0.80 is considered as very good.

The RAVD, on the other hand, quantifies the difference between the segmented and ground truth volumes. It is calculated as:

$$RAVD=\frac{\begin{array}{c}\textit{volume}\,\textit{segmentation}\\{}-\textit{volume}\,\textit{ground}\,\textit{truth}\end{array}}{\textit{volume}\,\textit{ground}\,\textit{truth}}\times 100{\%}$$

An RAVD of 0% indicates perfect alignment, and 100% indicates no overlap. For both metrics, we reported the mean, standard deviation, and 95% confidence intervals. Statistical analysis was conducted in Python (version 3.12) using additional packages such as Collections, Scikit-learn [18], Statsmodels [19], and SciPy [20].

Additionally, in order to investigate the potential of our model in detecting tumors or retinal detachment, we evaluated its performance using the Area Under the Receiver Operating Characteristic (AUROC) curve.

Additionally, in order to investigate the potential clinical use of our model in the measurement of the volumes of tumor and retinal detachment, we calculated the Intraclass Correlation Coefficient (ICC) between the automated measurements obtained from our model and the manual measurements obtained from our manual segmentation.

## Results

### Study Population

The final dataset consisted of 117 orbital MRI scans. The average patient age was 59 years, with a standard deviation of 15.2 years, and there was a slightly higher proportion of male patients (64, 54.7%) compared to female patients (53, 45.3%). The study primarily focused on patients with tumors, accounting for 110 cases (94.0%), while 7 patients (6.0%) had alternative conditions, such as endocrine orbitopathy. Regarding tumor classification, 101 patients were diagnosed with uveal melanoma (86.3% of total patients, 91.8% of tumor patients), making it the predominant tumor type in the dataset. A small portion of patients (9 patients, 7.7% of total patients and 8.2% of tumor patients) had other tumors such as lymphoma, hemangioma and meningioma.

Retinal detachment was present in 84 patients (71.8% of total patients, 76.4% of tumor patients). Among the tumor cases, 13 patients exhibited extraocular growth (11.1% of total patients and 11.8% of tumor patients), indicating possible complications or advanced stages of disease (Table 1).

**Table 1:** Population Characteristics

Paramter	Value	Percentage
Total Patients	117	100%
Gender (Male/Female)	64/53	54.7%/45.3%
Age (mean ± standard deviation)	59 ± 15.2	–
Patients with Tumors	110	94.0%
Patients without Tumors	7	6.0%
Uveal Melanoma	101	86.3% (of total)/91.8% (of tumor patients)
Other Tumors	9	7.7% (of total)/8.2% (of tumor patients)
Tumor with Extraocular Growth	13	11.1% (of total)/11.8% (of tumor patients)
Retinal detachment	84	71.8% (of total)/76.4% (of tumor patients)

### Deep Learning-based Segmentation

The loss-functions curves of the training set and the DSC of all five models are reported in Supporting Information Fig. 6. ANOVA testing revealed no significant effect of the fold number on the Dice Similarity Coefficient (DSC) across all segmented orbital structures. Specifically, the results showed no significant differences in DSC values for the eye (F = 0.318, *p* = 0.866), optic nerve (F = 0.846, *p* = 0.499), tumor (F = 0.971, *p* = 0.428), or retinal detachment (F = 0.910, *p* = 0.461). These findings indicate that the model’s segmentation performance remained consistent across cross-validation folds, supporting the robustness and stability of the training procedure.

### Results: Segmentation Performance

**Fig. 2:**
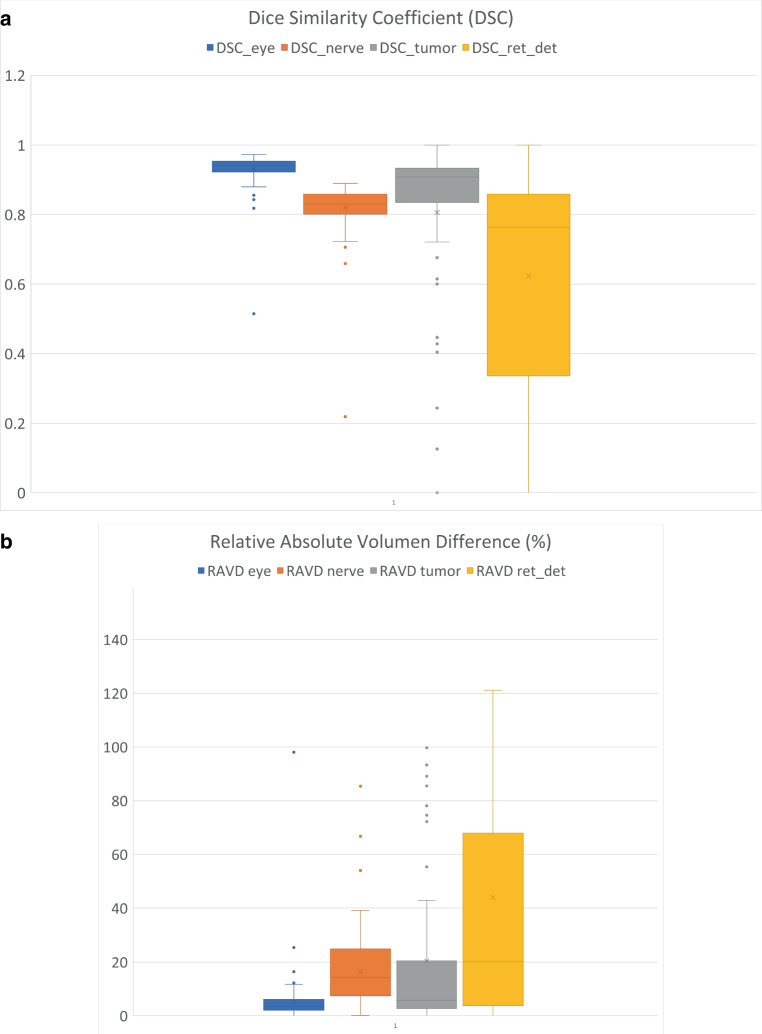
Boxplots comparing dice similarity coefficient (**a**) et relative absolute volume difference (**b**) across different structures

**Fig. 3:**
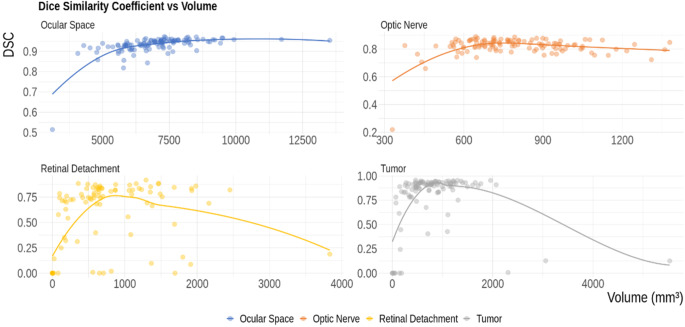
Association between DSC and Volume for the classes analyzed. The ocular space and optic nerve have a DSC consistently higher than 0.8, which is considered very good. Only one subject, with very low volumes due to an extended tumor and retinal detachment, resulted in a lower DSC. For retinal detachment, a logarithmic increase of DSC with volume can be seen, with retinal detachments larger than 300 mm^3^ resulting in a DSC higher than 0.75. Moreover, some retinal detachments with volumes between 500 and 2000 mm^3^ resulted in poor DSC due to bleeding that caused misclassification as tumors. For tumors, the same logarithmic increase of DSC can be seen as with retinal detachment. Additionally, three outliers with large tumor volumes were poorly segmented, probably because they were poorly represented in the training set

**Fig. 4:**
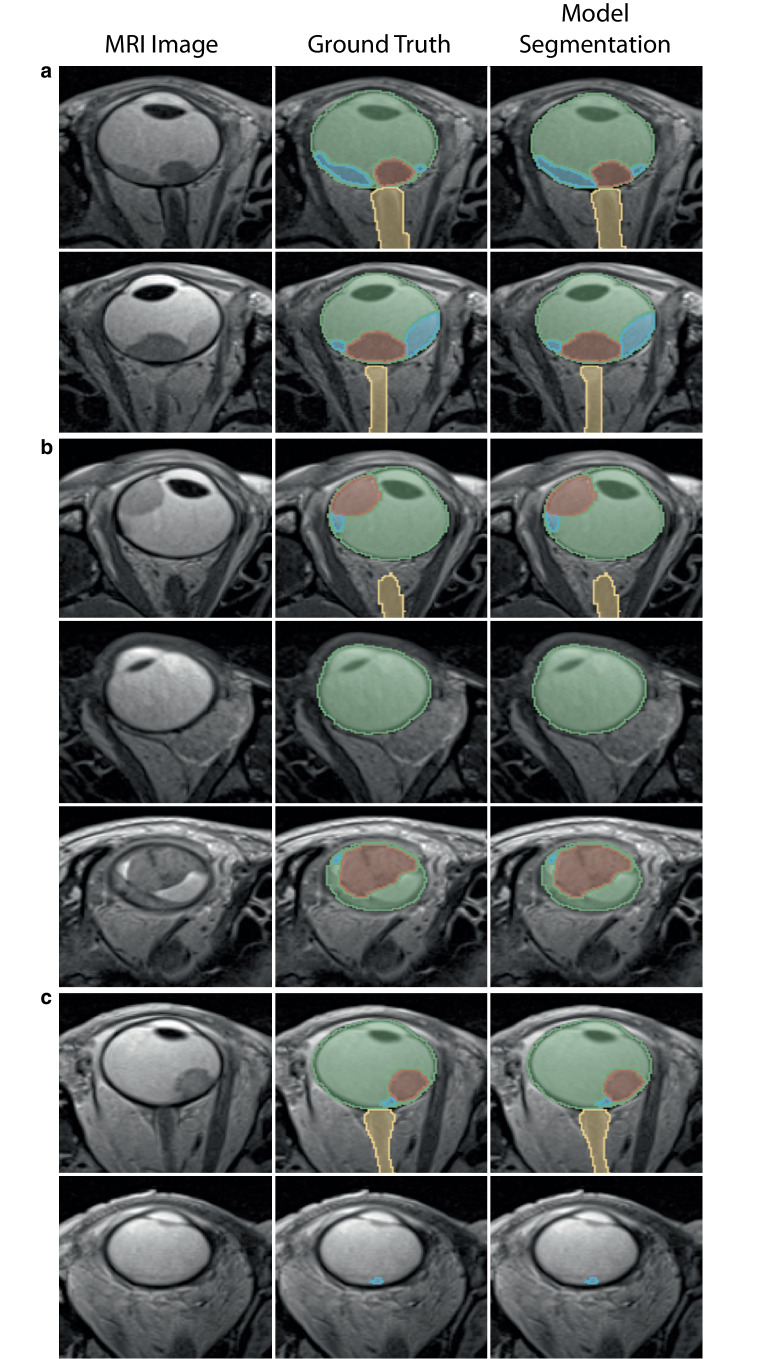
Sample images showing segmentation results. Generally, the U‑Net demonstrated a high segmentation performance across all four entities (ocular bulb, optic nerve, tumor and retinal detachment) (**a**, first and second row). For more challenging cases, the model showed slightly lower performance in segmenting extraocular tumors (**b**, second row) and retinal detachments, particularly when the intensity differences between the tumor and retinal detachment were minimal (**b**, third row). Additionally, the model struggled to identify retinal detachments when no tumor was present nearby (**c**, second row), indicating a bias influenced by surrounding areas. Even the small retinal detachment was identified as such when there was a tumor nearby (**c**, first row). Misidentification of signal artifacts, such as metal artifacts, as retinal detachments was also observed when a tumor was present (**b**, first row)

**Fig. 5:**
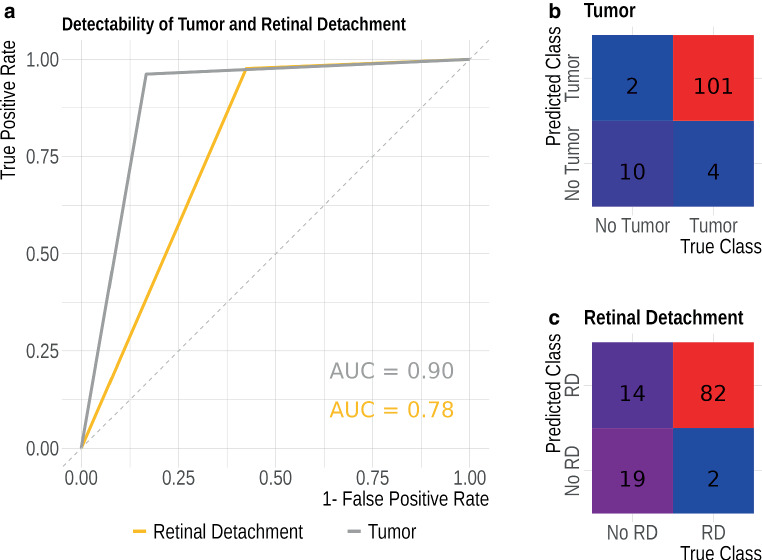
*Receiver Operating Characteristic (ROC) curves comparing the detectability of uveal melanoma tumors and associated retinal detachment. *The gray curve represents tumor detectability with an area under the curve (AUC) of 0.90, indicating high diagnostic performance. The yellow curve represents retinal detachment detectability with an AUC of 0.78, suggesting lower—but still significant—performance

**Fig. 6:**
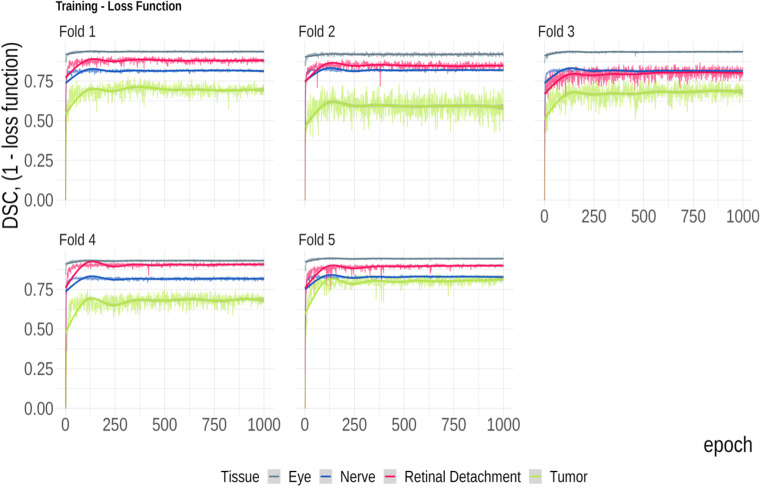
Training loss function across the folds and the training epochs shows that in some folds, the tumor loss function is lower, likely due to the underrepresentation of extraocular tumors. In other cases, the loss function of both retinal detachment and tumor shows higher variance. This is attributed to bleeding in the detachment, making the intensities of the two classes similar and resulting in part of the retinal detachment being misclassified as a tumor

U‑Net achieved a DSC value of 0.931 ± 0.047 for ocular bulb, 0.820 ± 0.069 for optic nerve, 0.788 ± 0.273 for tumor, and 0.550 ± 0.342 for retinal detachment. The mean RAVD were 5.04% for ocular bulb, 16.6% for optic nerve, 20.39% for tumor, and 44% for retinal detachment. All metrics are shown in Table 2 and Fig. 2. (Figure 3, 4 and 5).

**Table 2:** Performance metrics

Metric		Ocular bulb	Optic Nerve	Tumor	Retinal detachment
*DSC*	*Mean* *±* *SD*	0.931 ± 0.047	0.820 ± 0.069	0.788 ± 0.273	0.550 ± 0.342
	*95% CI*	0.92–0.94	0.81–0.83	0.74–0.84	0.48–0.62
*RAVD*	*Absolute* *value*	5.04%	16.6%	20.39%	44%
	*95% CI*	3.32–6.76	14.2–19	14.62–26.17	25.15–62.85
*AUC*	*Value*	–	–	0.90	0.78
*ICC*	*Value*	0.94	0.57	0.51	0.67
	*95% CI*	0.91–0.95	0.44–0.68	0.36–0.63	0.53–0.77

Segmentation performance. *SD* Standard Deviation, *95% CI* 95% Confidence Interval, *DSC* Dice Similarity Score (higher values indicate better performance), *RAVD* Relative Absolute Volume Difference (lower values indicate better performance)

The Area Under the Curve (AUC) values for our model were optimal (AUC = 0.90) in detecting tumors, whereas they were good (AUC = 0.78) in detecting retinal detachment.

The Intraclass Correlation Coefficient (ICC) values for our model were excellent for the ocular bulb. However, for other structures, the ICC values were acceptable but lower, with values of 0.57 for the optic nerve, 0.51 for tumor, and 0.67 for retinal detachment.

## Discussion

Orbital segmentation has significant clinical applications across patient care. Surgically, it provides precise visualization of complex structures, enhancing navigation-guided procedures [26]. In ocular oncology, it enables accurate tumor volumetry and improved distinction between neoplastic and healthy tissue, optimizing radiation planning and treatment monitoring [27]. For retinal detachments, automated segmentation quantifies extent and assists in treatment planning and follow-up [28]. In chronic conditions like endocrine orbitopathy, it objectively measures changes over time, allowing better assessment of therapeutic response [29]. These capabilities collectively improve clinical decision-making and patient care quality.

In this study, we successfully implemented a deep learning framework for the segmentation of orbital structures on MRI, including the ocular bulb, optic nerve, tumors, and retinal detachments. The achieved DSC scores for the ocular bulb and optic nerve (each > 0.8) highlight the model’s performance for these anatomical structures.

Conventional segmentation techniques have historically played a central role in ophthalmic imaging, particularly for structures such as the optic disc and retina. These methods—including edge detection, thresholding, and region-growing algorithms—are well-established and relatively easy to implement, especially in low-resource settings. However, they are often sensitive to noise, require manual parameter tuning, and show limited adaptability to variations in image quality or new datasets, with reported accuracies typically ranging between 70–85%. In contrast, deep learning-based approaches have demonstrated superior performance, with segmentation accuracies often exceeding 90%. These AI-driven models are highly adaptive, capable of learning from large datasets, and more robust to image variability, offering potential for real-time clinical deployment. Nevertheless, they come with challenges such as high computational demands, risks of overfitting when trained on small datasets, and reduced interpretability. The transition from conventional to AI-driven methods marks a significant advancement in the field, enabling more precise and scalable analysis of ophthalmic structures [21].

Our results align with the current state of research while extending beyond it in several aspects. The high segmentation accuracy for the ocular bulb corresponds to findings from other research groups such as Strijbis et al. [22], who achieved a DSC value of 0.97 ± 0.01, and Nguyen et al. [23] with a DSC of 88.3%. This agreement suggests a convergence toward optimal methodologies for well-defined anatomical structures.

In contrast to the relatively simple segmentation of the ocular bulb, the precise delineation of tumors presents a greater challenge. We achieved a DSC between 0.7 and 0.8, which is slightly lower compared to Strijbis et al. [22] with 0.84 ± 0.23. However, Strijbis et al. used three different imaging modalities on a smaller sample of 30 patients, while our method is based exclusively on MRI data and is thus more clinically practicable. Compared to other approaches such as those by Ciller et al. [24], who achieved only 64.9 ± 6.3%, our model shows clear superiority. This variance in the performance of different algorithms reflects the challenge of precisely segmenting variable pathological structures as discussed in recent literature [31].

For optic nerve segmentation, we achieved slightly better results (DSC > 0.8) than Nguyen et al. [25] with 81.9%. This improvement could be attributed to the architecture of nnU-Net, which according to Isensee et al. [32] is particularly effective in detecting elongated anatomical structures. Optic nerve segmentation plays a crucial role in the quantitative analysis of diseases such as idiopathic intracranial hypertension and neuromyelitis optica, as highlighted by Chen et al. [33].

Regarding retinal detachments, there are few comparative studies, with Strijbis et al. [22] achieving better results with a DSC of 0.79 than our model. This discrepancy could be explained by the difficulty of segmenting structures with small volume and variable morphology as described in the literature [34]. The clinical relevance of precise segmentation of retinal detachments was emphasized by Sharma et al. [35], who showed that volumetric measurements can play a crucial role in treatment decisions.

A notable observation in our study is the varying performance depending on the signal intensities of the tumors. The more similar the T2 intensities between tumors and retinal detachments were, the more difficult and error-prone the segmentation became, while more precise segmentation results were achieved in cases with clearly distinguishable intensities. The T2 signal characteristic significantly influences segmentation accuracy, a phenomenon also observed in brain tumor segmentation studies [36]. This underscores the necessity of training deep learning models with a balanced distribution of different signal intensities.

The development and validation of deep learning algorithms for orbital imaging is in line with the broader trend toward automation of radiological tasks. The choice of network architecture is crucial: while earlier studies often used custom CNN architectures, the nnU-Net approach we used shows remarkable adaptability to various medical segmentation tasks. Isensee et al. [32] demonstrated that their self-configuring framework has won many biomedical segmentation challenges and achieved state-of-the-art performance across multiple datasets, often outperforming specialized architectures developed for specific tasks.

Our methodological limitations, particularly the reliance on a single radiologist and one resident for creating the ground truth, reflect a known challenge in medical image processing. Kumar et al. [37] demonstrated that integrating multiple independent annotations can improve the generalizability of segmentation algorithms. Future studies should therefore implement a multi-reader approach as recommended by Joskowicz et al. [38], who highlighted the significant interobserver variability in manual contour delineation of structures in CT scans and the need for standardized consensus procedures. Another limitation is that the segmentation of the optic nerve and ocular bulb did not include pathological alterations, so the high segmentation performance would not automatically apply to cases with pathologies of the optic nerve or ocular bulb.

Our study has several methodological limitations. Most notably, the ground truth was established by a single radiologist and one resident, which underscores a common challenge in medical image annotation. As demonstrated by Kumar et al. [37], incorporating multiple independent annotations can significantly enhance the generalizability and robustness of segmentation algorithms. Future research should therefore adopt a multi-reader approach, as advocated by Joskowicz et al. [38], who emphasized the substantial interobserver variability in manual contouring of anatomical structures in CT scans and the importance of standardized consensus procedures. Additionally, our segmentation of the optic nerve and ocular bulb did not account for pathological alterations. As a result, the high segmentation performance observed may not generalize to cases involving optic nerve or ocular bulb pathologies.

The single-center nature of our study and the specialization of our center in uveal melanoma led to an overrepresentation of this relatively rare disease in our dataset (86.3% of total patients, 91.8% of tumor patients). Moreover, all patients have been examined only in one 3T MRI machine with specific acquisition parameters that have been adapted to the needs of the surgeons of our center. We consider these factors and the imbalanced distribution a significant limitation of our study, as it could restrict the generalizability of our results to diverse pathological phenotypes. Nalepa et al. [39] reviewed various augmentation techniques that can improve segmentation accuracy when working with limited datasets. During the development of this study, some augmentation strategies for the poorly represented classes were implemented, but they did not yield an improvement in the results. The release of our pretrained models as open-data resources promotes external validation and further development.

While our model demonstrates lower segmentation performance for retinal detachment compared to other structures, it still offers significant clinical value. Further analysis revealed that the model achieves a detection AUC of 0.78 for retinal detachment, indicating reasonably good performance in identifying its presence. Importantly, in current clinical practice, retinal detachment is typically noted qualitatively by radiologists rather than being precisely segmented or quantified. Therefore, a model that can reliably detect its presence—even without perfect segmentation—can still provide meaningful support in clinical decision-making.

Moreover, it is important to emphasize that treatment pathways for uveal melanoma vary considerably. A substantial proportion of patients undergo proton beam radiotherapy rather than surgery. In these cases, accurate segmentation of the ocular bulb and tumor volume—as provided by our model—is highly relevant for pre-radiation planning. Thus, while segmentation of retinal detachment remains a limitation, the model still delivers clinically actionable information that supports treatment planning in a significant subset of patients.

In summary, our nnU-Net-based model has demonstrated robust performance in the segmentation of orbital structures, highlighting its strong potential for clinical application. The observed challenges in the segmentation of tumors and retinal detachments offer important directions for future research. Performance may be further improved with larger and more diverse datasets, including multimodal imaging. As part of this study, we are releasing a model trained on the full dataset, which can be fine-tuned in future investigations. To ensure broader clinical applicability, future work should include multi-center validations across institutions using varied MRI protocols to assess the model’s generalizability and translational potential. Our model contributes to the development of next-generation AI-assisted segmentation tools that may significantly enhance precision medicine in ophthalmology and neuroradiology.

## Figures and Tables

**Fig. 7:**
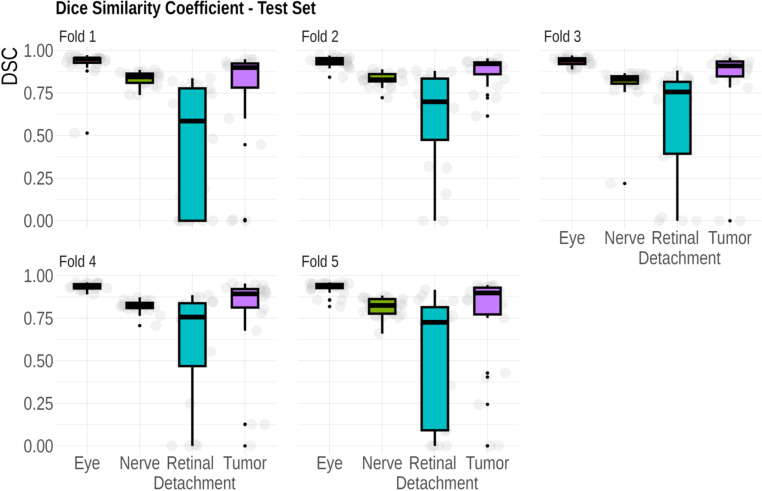
Segmentation performance as DSC for the individual folds of the five-fold cross-validation shows that the DSC for eye and nerve are generally high. However, the average DSC for tumor and retinal detachment are lowered due to a few cases with DSC values very low or around zero. For tumors, this may be due to some underrepresented extraocular tumors. In other cases, bleeding in the retinal detachment makes the intensity resemble that of the tumor, causing misclassification and lowering the DSC of both classes

## Data Availability

nnUNet pretrained models are available on OSF.io. Exemplificatory code is available on GitHub (10.17605/OSF.IO/SY6JB)
